# Sensory nerves in the spotlight of the stem cell niche

**DOI:** 10.1002/sctm.20-0284

**Published:** 2020-10-28

**Authors:** Caroline C. Picoli, Alinne C. Costa, Beatriz G.S. Rocha, Walison N. Silva, Gabryella S.P. Santos, Pedro H.D.M. Prazeres, Pedro A.C. Costa, Anderson Oropeza, Rodrigo A. da Silva, Vasco A.C. Azevedo, Rodrigo R. Resende, Thiago M. Cunha, Akiva Mintz, Alexander Birbrair

**Affiliations:** ^1^ Department of Pathology Federal University of Minas Gerais Belo Horizonte Minas Gerais Brazil; ^2^ Department of Dentistry University of Taubaté Taubaté São Paulo Brazil; ^3^ Cellular and Molecular Genetics Laboratory, Department of Genetics, Ecology and Evolution Federal University of Minas Gerais Belo Horizonte Minas Gerais Brazil; ^4^ Department of Biochemistry and Immunology Federal University of Minas Gerais Belo Horizonte Minas Gerais Brazil; ^5^ Department of Pharmacology University of São Paulo Ribeirão Preto São Paulo Brazil; ^6^ Department of Radiology Columbia University Medical Center New York New York USA

**Keywords:** genetic depletion, mesenchymal stem cells, microenvironment, niche, sensory nerves

## Abstract

Niches are specialized tissue microenvironments that control stem cells functioning. The bone marrow mesenchymal stem cell niche defines a location within the marrow in which mesenchymal stem cells are retained and produce new cells throughout life. Deciphering the signaling mechanisms by which the niche regulates stem cell fate will facilitate the use of these cells for therapy. Recent studies, by using state‐of‐the‐art methodologies, including sophisticated in vivo inducible genetic techniques, such as lineage‐tracing Cre/loxP mediated systems, in combination with pharmacological inhibition, provide evidence that sensory neuron is an important component of the bone marrow mesenchymal stem cell niche. Strikingly, knockout of a specific receptor in sensory neurons blocked stem cell function in the bone marrow. The knowledge arising from these discoveries will be crucial for stem cell manipulation in the future. Here, we review recent progress in our understanding of sensory nerves biology in the stem cell niche.

## Graphical abstract

Schematic illustration summarizing the findings from genetic deletion of EP4 specifically from sensory nerve fibers in the bone marrow mesenchymal stem cell niche.

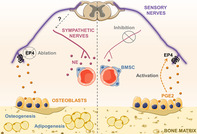

**Significance statement**

The understanding of the neural regulation in the stem cell niche in the bone marrow and in other organs still remains limited, and the complexity of these interactions in different microenvironments should be elucidated. This article reviews recent progress in the understanding of sensory nerves biology in stem cell niches. Recent studies provide evidence that sensory neurons are important components of the mesenchymal stem cell niche. The emerging knowledge from this research will be important for the treatment of several disorders.

## 1 INTRODUCTION

### 1.1 Bone marrow mesenchymal stem cells (BMSCs)

Adult endogenous stem cells are fundamental for maintaining tissue homeostasis due to their extraordinary capacity to form specialized cell populations in a coordinated way according to the needs of the organism.^1^ Mesenchymal stem cells were first discovered within the bone marrow.^2^ Subsequent studies have identified mesenchymal stem cells in various other adult tissues.^3^ BMSCs are characterized as postnatal self‐renewing multipotent stem cells, forming all skeletal tissues.^2^, ^4^ In culture, these cells can form a clonal progeny of transplantable cells, equal to the one that generated them.^5^ After transplanted in vivo, BMSCs can form bone organoids.^6^ A single BMSC is a bona fide stem cell as it can initiate a clonal population in vitro, which then may create a full organoid in vivo with transplantable BMSCs, being self‐renewing and multipotent.^2^, ^7^ BMSCs are also defined as skeletal stem cells as they can be located within the skeleton, able to give rise to various skeletal tissues, and have an innate capacity to start a recapitulation of bone organogenesis in vivo.^8^, ^9^, ^10^, ^11^ BMSCs are essential for the development, lifelong turnover, and regenerative ability of bones in our organism.^12^, ^13^ The ability of mesenchymal stem cells derived from variable sources to repair tissues placed them in the center of attention of numerous groups due to their promising potential in regenerative medicine for multiple disorders.^14^, ^15^ Therefore, in the last two decades, it became clear that understanding the biology of these cells may lead to the treatment of several diseases.

### 1.2 Stem cells and their niches

Accurate regulation over stem cell differentiation is crucial for appropriate tissue homeostasis and organogenesis.^16^ Stem cells occupy particular microenvironments, also termed niches,^17^ which keep them in an undifferentiated and self‐renewing state. Defining and understanding the mechanisms that restrict niche signaling exclusively to stem cells is crucial to determine how stem cells undergo self‐renewal while their progeny differentiate. Extensive studies in a variety of tissues have highlighted the importance of the microenvironment in modulating stem cell behavior, including skin,^18^ intestine,^19^ stomach,^20^ skeletal muscle,^21^ bone marrow,^22^ liver,^23^ brain,^24^, ^25^ and others.^26^, ^27^ Despite significant progress made in our knowledge of which signals foster stem cell quiescence or activation, some constituents of stem cells niches remain unrevealed to date. This is due to the complexity of tissue microenvironment content and its dynamics. Understanding the role of niche components in stem cell behavior is vital for our knowledge of organ homeostasis and disease, and to fully exploit stem cell therapeutic potential.

### 1.3 BMSC niche

Although the precise location of the BMSC niche has not been determined so far, several studies suggest that MSCs reside in perivascular sites, associated with blood vessels.^28^ Therefore, MSCs have been compared to pericytes.^29^, ^30^, ^31^, ^32^, ^33^, ^34^, ^35^, ^36^, ^37^, ^38^, ^39^, ^40^, ^41^, ^42^, ^43^, ^44^, ^45^ Nevertheless, whether these two cell types correspond to the same cell is not clear yet.^11^, ^46^

BMSCs maintenance within the adult bone is essential for skeletal homeostasis and reconstruction after damage.^47^ BMSCs are often in a quiescent state, and extrinsic factors from their medullar niche can activate their self‐renewal, proliferation, or differentiation. Examination of the BMSC microenvironment in the bone marrow revealed that BMSC behavior is markedly affected by interactions with cellular components of this local niche, both directly, by physical contact, or indirectly, by ligation of secreted soluble molecules.^48^ Multiple local signaling cues can influence BMSCs fate, such as interleukins,^49^, ^50^ chemokines,^51^ Wnt ligands,^52^, ^53^ FGF2,^54^ and others.^48^, ^55^ Oxygen also may be a key regulatory factor in the BMSC niche.^56^ Perturbations in the architecture of extracellular matrix constituents in the bone marrow influence BMSC behavior.^57^, ^58^, ^59^, ^60^, ^61^ Moreover, mechanical stimuli from the microenvironment can direct BMSC lineage specification.^57^, ^58^, ^62^, ^63^, ^64^ Several groups are trying to identify crucial components of the BMSC niche, and how these key components regulate BMSC behavior and fate. The BMSC niche composition is complex and heterogeneous. Besides BMSCs which can regulate themselves,^49^, ^65^ an array of distinct cell types support and communicate with BMSCs. These include endothelial cells,^59^, ^60^, ^66^, ^67^ osteoblasts,^68^ osteocytes,^50^, ^69^ chondrocytes,^70^ hematopoietic progenitors,^51^ fibroblasts,^71^ immune cells,^72^, ^73^ and others.^74^, ^75^ As innervations are also present within the bone marrow, they became strong candidates for a role in the BMSC niche.^76^, ^77^, ^78^, ^79^

### 1.4 Nerves in the bone marrow

Nerves penetrating the bone marrow were described for the first time more than 50 years ago.^80^ As the bone marrow occupies spaces deep within our body and is encased by an outer hard compact bone, experimental assessments were initially difficult. Later studies confirmed these initial discoveries demonstrating that medullar innervations can signal and communicate with cells in the bone marrow.^81^ Innervations in the bone marrow are mostly associated with blood vessels.^80^, ^82^ The pattern of similar wiring of nerves and blood vessels is well established in other organs, and suggests that they support each other.^83^ Accordingly, it is possible that vascular and neuronal networks also have a functional connection within the bone marrow. Because of the perivascular location of BMSCs, the interaction of BMSCs with nerve projections is also likely.

So far, the sympathetic nerves were the most explored nerve fibers in the bone marrow. Innervations expressing tyrosine‐hydroxylase^79^ and Neuropeptide Y,^84^ an abundant sympathetic co‐transmitter, were detected. Examination of the bone marrow from Neuropeptide Y knockout mouse model revealed a decrease in the number of BMSCs, indicating that Neuropeptide Y signaling may be important for BMSC maintenance.^85^ Genetic or pharmacological depletion of sympathetic signaling in the bone marrow triggers the expansion of BMSCs with reduced CFU‐F capacity, also impairing BMSCs' differentiation capacity.^7^, ^77^ The loss of sympathetic nerves also results in reduction of BMSC ability for hematopoietic stem cell maintenance.^7^, ^76^, ^77^, ^86^ Moreover, sympathetic denervation culminates in BMSCs mobilization from their niche to bone‐forming sites.^87^ Together, these findings identify that sympathetic innervations can regulate BMSC behavior in the bone marrow, and suggest that BMSCs' maintenance, proliferation, and differentiation are controlled by these nerve fibers.

### 1.5 Sensory nerves in the bone marrow

Sensory neuronal projections also penetrate the bone marrow microenvironment as abundant single or bundled fibers usually coupled with medullar blood vessels.^79^, ^88^ These fibers express calcitonin gene‐related peptide, substance P, and/or vasoactive intestinal peptide.^84^, ^88^, ^89^, ^90^, ^91^ In contrast to sympathetic innervations in the bone marrow,^77^ sensory nerves do not diminish with aging.^79^ Although the sensory nerves innervate the bone marrow and are located in close proximity to BMSCs, little is known about their role in the bone marrow microenvironment.

Dissecting the complex pathways leading to stem cells activation may be very challenging. Understanding whether sensory nerves are part of the BMSC niche, the signaling mechanisms by which they control the mesenchymal stem cell fate may be crucial for the success of clinical applications. In a recent article in *The Journal of Clinical Investigation*, Hu and colleagues demonstrated that sensory nerves regulate adult mesenchymal stem cells behavior in vivo via control over sympathetic nerves activity.^92^ Using state‐of‐the‐art techniques including sophisticated in vivo inducible genetic approaches, such as lineage‐tracing Cre/loxP mediated technologies, in combination with pharmacological approaches, microtomography, and confocal microscopy, the authors selectively eliminated sensory neurons to analyze their role in bone homeostasis. The authors specifically ablated sensory innervations genetically, by using Advillin‐Cre/TrkA‐floxed and Advillin‐Cre/iDTR mice, and pharmacologically, by capsaicin treatment. These experiments revealed that the absence of sensory neuronal projections promotes fat formation at the expense of osteogenesis in the adult bone marrow.^92^ Importantly, the number of BMSCs diminished in vivo, as well as their ability to differentiate into osteoblasts in vitro,^92^ suggesting that sensory innervations are essential for BMSCs maintenance in their niche.

**FIGURE 1:**
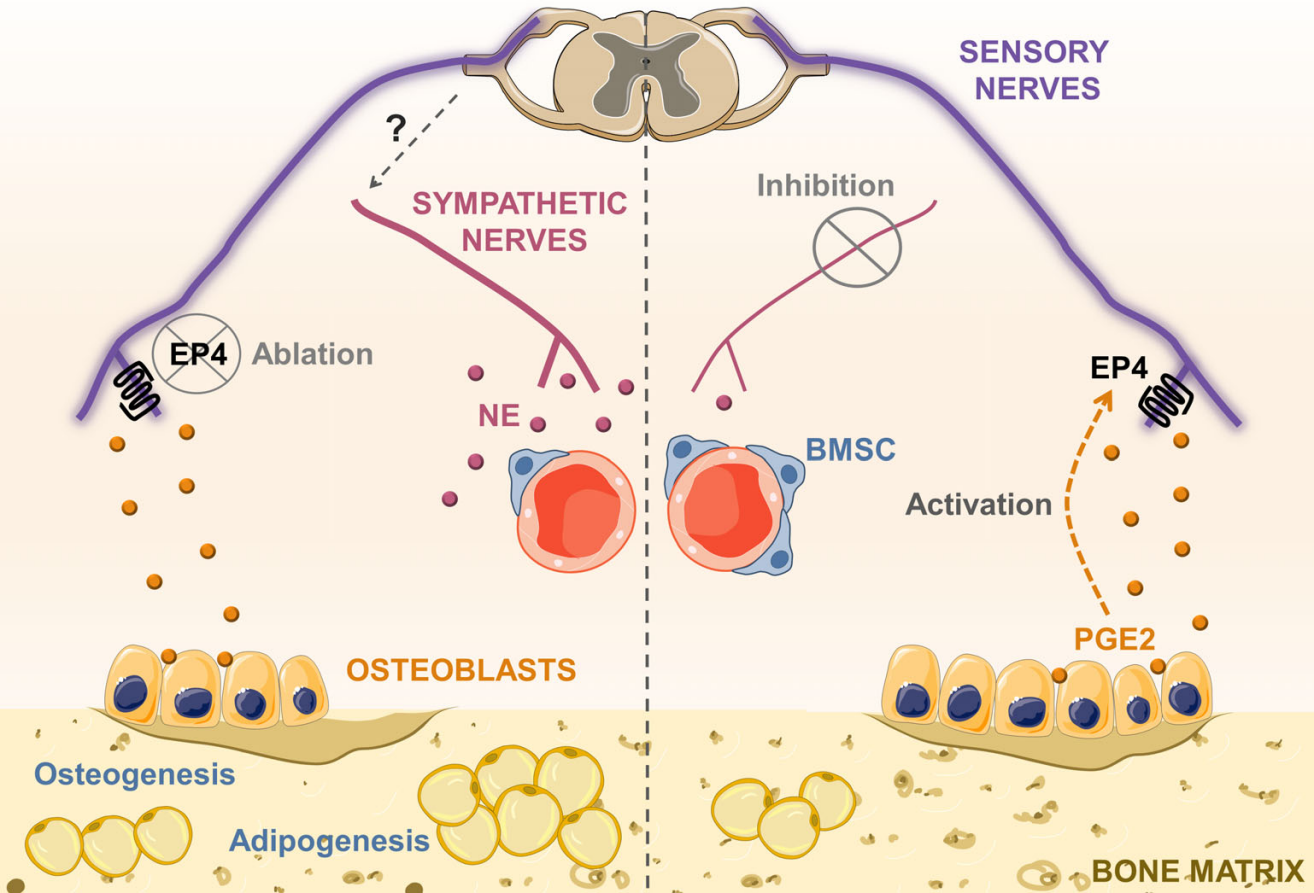
Schematic illustration summarizing the findings from genetic deletion of EP4 specifically from sensory nerve fibers in the bone marrow mesenchymal stem cell (BMSC) niche. Sensory fibers innervate the bone marrow BMSC niche. (Right) Sensory nerves maintain MSC number in the bone marrow, which differentiate normally into osteoblasts. (Left) In contrast, genetic ablation of EP4 from sensory innervations reduces the number of BMSCs, likewise their capacity to self‐renew and to differentiate into the osteogenic lineage. This leads to inhibited osteogenesis and induced adipogenesis^92^

It is well established that prostaglandins play essential roles in bone metabolism.^93^ Amid prostaglandins, prostaglandin E_2_ (PGE_2_) induces bone formation.^94^ Strikingly, genetic deletion of prostaglandin E_2_ receptor (EP4) specifically from sensory nerve fibers, by using Advillin‐Cre/EP4‐floxed mice, inhibited osteogenesis and induced adipogenesis.^92^ Moreover, similarly to what was observed after sensory nerves depletion, BMSCs number declined in the bone marrow, along with their capacity to self‐renew and to differentiate into the osteogenic lineage^92^ (Figure 1). Genetic ablation of the prostaglandin receptor from other cells present in the bone marrow microenvironment (osteoblasts or BMSCs) did not present alterations in the bone or in BMSC behavior.^92^ Overall, these findings imply that EP4 receptor in sensory nerves is crucial for BMSC behavior control in the bone marrow.

Hu and colleagues also showed, by using Ocn‐Cre/COX2‐floxed mice and pharmacological inhibition, that osteoblasts are the source of PGE_2_ which acts on sensory nerves that consequently affect BMSC behavior.^92^ Remarkably, Hu and colleagues demonstrated that mechanical loading induces osteoblasts to produce PGE_2_, linking a physiological state to stem cell regulation. Furthermore, the authors demonstrated that blockage of β_2_ adrenergic signaling abolishes bone and BMSC changes visualized in Advillin‐Cre/EP4‐floxed mice. These data suggest that sensory innervations affect BMSCs by deactivating sympathetic signaling in the bone marrow. Additionally, as BMSCs are pivotal in bone regeneration, Hu and colleagues examined a mouse model of femur fracture in which EP4 was ablated from sensory nerves. This examination unveiled that EP4 in sensory nerve fibers is vital for osteoinduction of BMSCs for bone fracture healing. Taken together, these results indicate that sensory nerves are fundamental components of the BMSC niche in the bone marrow which regulate BMSC functions. This study reveals a new component of the BMSC niche: sensory nerves; linking physiological states to BMSCs activation. Here, we discuss the findings from this work, and evaluate recent advances in our understanding of the bone marrow microenvironment and sensory nerves biology.

## 2 PERSPECTIVES/FUTURE DIRECTIONS

### 2.1 Specificity of transgenic mouse models

Hu and colleagues explored tissue‐specific null mutant mouse models (Advillin‐Cre/EP4‐floxed, Ocn‐Cre/COX2‐floxed, Ocn‐Cre/EP4‐floxed, and Lepr‐Cre/EP4‐floxed mice).^92^ Some caveats need to be given due consideration when using such systems, including insufficient gene knockdown and compensatory pathway upregulation. Thus, the analysis of the expression level of the gene being deleted as well as of other genes that may be affected will clarify this point. Moreover, gene ablation that occurs in the germline can culminate in developmental compensatory mechanisms.^95^, ^96^, ^97^ Models with inducible time and tissue‐specific gene ablation would overcome potential physiological compensatory processes that can alter the true function of a specific gene.^98^ Such strategies are being used to study the role of individual proteins in distinct pathophysiologic conditions.^99^, ^100^, ^101^, ^102^, ^103^, ^104^, ^105^, ^106^ Thus, this may be addressed by analyzing bone marrow from Advillin‐CreER/EP4‐floxed mice in which EP4 deletion in sensory nerve fibers can be temporally controlled.

The main findings from this work are based on the data obtained from Advillin‐Cre mice.^92^ Note, however, that expression of advillin is not restricted to peripheral sensory neurons that innervate the bone marrow. Thus, in Advillin‐Cre/EP4‐floxed mice, EP4 is also eliminated from sensory nerves in several other tissues, besides the bone. Therefore, it remains to be explored whether BMSC phenotype in this mouse model is due to EP4 deletion that happens specifically in the bone. Moreover, it was recently discovered that advillin is also expressed in the peripheral neuronal projections innervating the vasculature coming from sympathetic, parasympathetic, and enteric neurons.^107^ Interestingly, even some non‐neuronal cells may be targeted in these mice, that is, Merkel^107^, ^108^ and Tuft^109^ cells. Hence, it is possible that some of the effects observed in BMSCs in Advillin‐Cre/EP4‐floxed mice are not due to sensory neurons exclusively. To clarify whether EP4 is eliminated from any other medullar components, the bone marrow from Advillin‐Cre/EP4‐floxed/TdTomato mice should be examined in which all components from which EP4 is being deleted will be labeled. Furthermore, mouse models more specific to sensory nerves can be used for comparison, such as Nav1.8‐Cre mice.

### 2.2 Mechanism by which sensory nerves affect BMSCs

Cao's group data revealed that sensory nerves regulate BMSC behavior in the bone marrow.^92^ Yet it remains uncertain whether this happens by an indirect mechanism via BMSC niche components or by directly acting on BMSCs. The authors suggest that this regulation is via sympathetic nervous system, based on pharmacological inhibition with β adrenergic antagonists.^92^ As beta‐blockers may have off‐target side effects,^110^ a direct evidence that sympathetic neurons are the main/only functionally important path for the effect of sensory nerves on BMSCs is still needed. To verify the role of sympathetic input on sensory nerves’ function within the BMSC niche, genetic sympathectomy may be performed in combination with sensory nerve ablation. This may be achieved by using TH‐Cre/iDTR mice, in which diphtheria toxin injection causes apoptosis of peripheral sympathetic nerves.^111^

Interestingly, in another recent work from the same group, Chen and colleagues suggest that sensory nerves' effect on bone formation is through the hypothalamus.^112^ The authors show that, in mice stimulated by PGE2, CREB is phosphorylated in the hypothalamus, and this is inhibited by knockout of EP4 receptors in sensory nerves. Albeit CREB phosphorylation in the hypothalamus may affect sympathetic nerves,^113^ no direct evidence that sensory nerves act on MSCs via hypothalamus is available yet. Therefore, it remains an exciting open question whether/how sensory nerves affect sympathetic nerves function in the BMSC niche. In addition to experiments in transgenic mouse models, single cell transcriptomic analysis will help us understand the central and peripheral nervous system involvement on the role of sensory nerves within the BMSC niche.

Importantly, sensory nerves may be stimulated to release a variety of neuropeptides, such as calcitonin gene related peptide, vasoactive intestinal peptide, tachykinins (Substance P, neurokinin A, neurokinin B), and others.^114^, ^115^, ^116^, ^117^ Whether sensory nerves act directly on bone marrow BMSCs through any of these mediators remains unknown, and should be addressed in future studies.

### 2.3 Sensory nerves role in other stem cell niches

After Schofield Raymond proposed the notion of stem cell niche for hematopoietic stem cells,^17^ this concept was adopted for several other stem cells. Stem cell specialized microenvironments have been described for BMSCs,^118^ neural stem cells,^119^, ^120^ satellite cells,^21^ hair follicles stem cells,^121^ intestinal stem cells,^122^ gonadal stem cells,^123^ liver stem cells,^23^ and others. Cao's group indicates sensory nerves for the first‐time as a component of a stem cell niche.^92^ As bone marrow BMSCs, key components of hematopoietic stem cell niche, are affected by sensory nerves, hematopoietic stem cells will be as well. Nonetheless, it will be important to examine whether sensory nerves can regulate hematopoietic stem cells also independently from their effect on BMSCs. As sensory nerves innervate multiple organs,^124^ future studies should explore the role of these nerves in stem cell niches of other organs.

**FIGURE 2:**
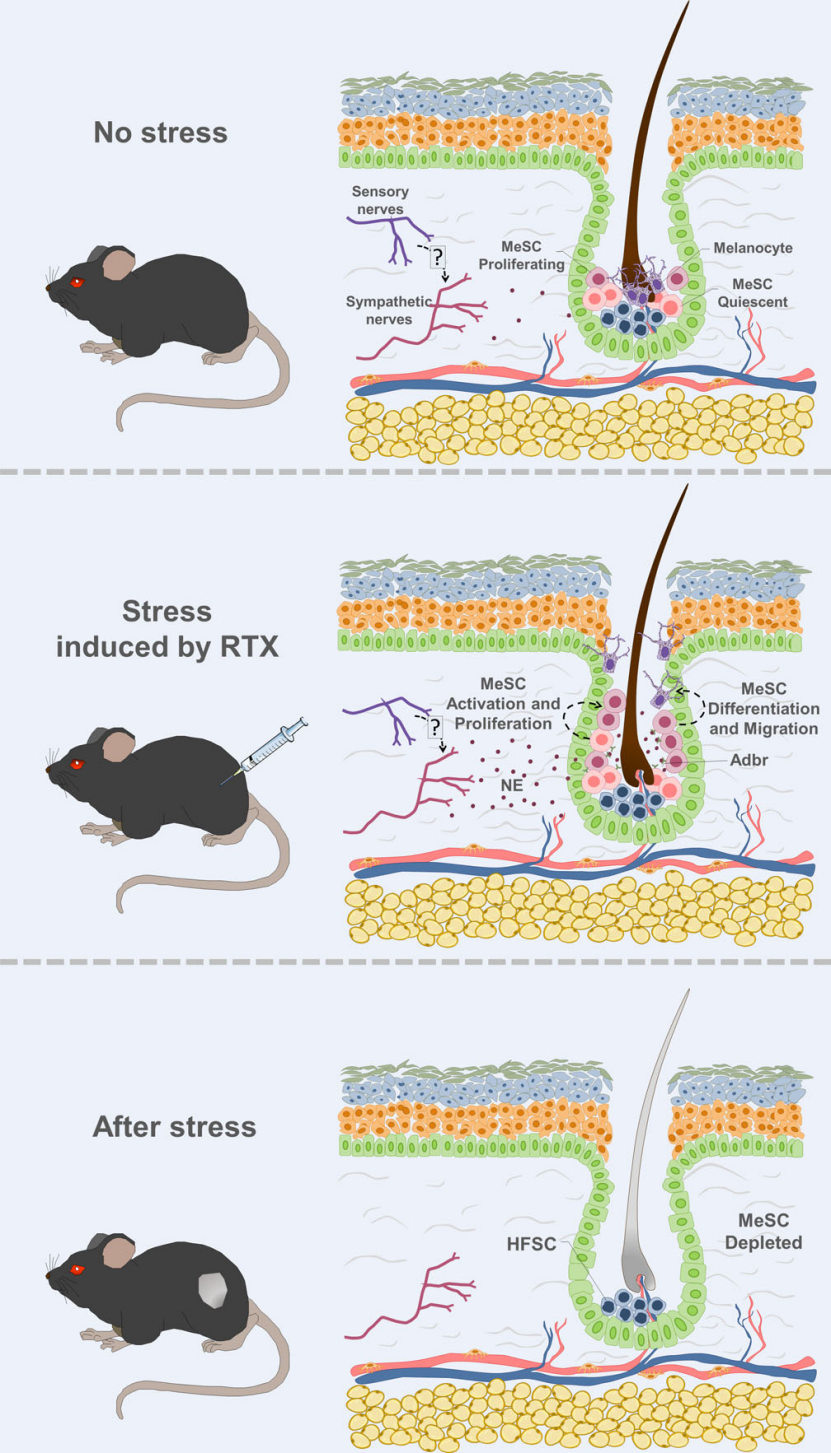
Sensory nerves role in the hair follicle melanocyte stem cell (MeSC) niche. Two main stem cell populations are present in the hair follicle bulge: MeSCs and hair follicle stem cells (HFSCs). MeSCs generate new pigmented hair. Zhang and colleagues revealed that hyperactivation of sympathetic nerves leads to MeSCs activation, proliferation, and consequently elimination from their niche, leading to hair discoloration.^126^ Interestingly, resinoferatoxin (RTX), which causes sensory nerve denervation as well, was used to induce stress. Adrb2, β2‐adrenergic receptor; NE, norepinephrine

One such organ is the skin, largely innervated by sensory nerve fibers.^125^ Interestingly, a recent study showed that hyperactivation of the sympathetic nervous system leads to reduced number of melanocyte stem cells in their dermal niche^126^ (Figure 2). These stem cells reside in the hair follicle microenvironment.^127^ Zhang and colleagues used Cre/loxP mediated technologies in combination with chemogenetics to evaluate stress effect on melanocyte stem cells in the hair follicle niche. These experiments revealed that hyperactivation of sympathetic nerves leads to melanocyte stem cells activation, proliferation, and consequently elimination from their niche.^126^ Interestingly, resinoferatoxin, which causes sensory nerve denervation as well,^128^ was used to induce stress. This suggests that possibly sensory nerves also may have a role in the hair follicle melanocyte stem cell niche.

Curiously, a recent study using elegant techniques including sophisticated lung transplants in combination with in vivo lineage‐tracing technologies, identified surprisingly a niche for hematopoietic stem cells in the lungs.^129^, ^130^ As these organs are densely innervated by sensory nerves,^131^ the role of sensory innervations in the pulmonary hematopoietic stem cell niche should be explored.

Interestingly, Cao's group showed that mice with sensory nerves genetic depletion present also impaired hepatic regeneration after hepatectomy, suggesting that sensory nerves compose the liver stem cell niche as well.^112^ Notably, the mechanism by which sensory nerves are activated in the liver is different from what happens in the bone marrow, as deletion of EP4 receptor in sensory nerves did not alter hepatic regeneration.^112^ During fetal development, hematopoietic stem cells are located in the liver from where they later migrate through the bloodstream to the bone marrow preceding birth, where they remain throughout the adult life.^132^, ^133^, ^134^, ^135^ Their expansion in the fetal liver is dependent of portal vessel‐associated hepatic mesenchymal stem cells.^132^ It will be interesting to explore whether sensory nerves also regulate hepatic mesenchymal stem cells in the fetal liver, and consequently hematopoietic stem cells.

### 2.4 Sensory nerve involvement in BMSCs' other functions

The most well‐established functions of BMSCs, based on which they were named, are related to their capacity to differentiate in multiple cell types, replacing damaged cells.^136^ Notably, in addition to their regenerative activities, BMSCs have also been described to present immunomodulatory, immunosuppressive, and anti‐inflammatory characteristics.^137^, ^138^, ^139^ These capabilities are the basis for the medical exploration by numerous clinical trials of BMSCs in the therapy for inflammatory and immune disorders.^140^, ^141^, ^142^ BMSCs can affect the behavior of various immune cells, such as T cells, macrophages, and others.^143^, ^144^, ^145^ These interactions may grant homeostasis within the tissue.^146^, ^147^

In the last decade, several groups have focused their attention on exploring the immune‐modulating capacity of the peripheral nervous system. Recent studies have shown that sensory neurons are critical mediators of inflammatory processes in diverse tissues.^128^, ^148^, ^149^ Nevertheless, what are the cellular and molecular mechanisms by which sensory nerves regulate immune responses remain uncertain. The regulation of BMSCs behavior by sensory innervations brings the question whether stem cells are involved in immune regulatory effects of sensory neurons. Also, it will be important to investigate which cues derived from sensory nerves may affect BMSCs and how.

### 2.5 Sensory nerves heterogeneity

Hu and colleagues assume sensory nerves to be a homogeneous population in their study.^92^ However, sensory nerves have been shown to be heterogeneous, comprising various subtypes with distinct functions.^150^ Sensory neurons even within the same ganglia may differ in their embryonic origins.^151^

Classically, different sensory neurons have been divided based on specific molecular markers and cell‐body diameters. For instance, sensory neurons expressing neurotrophic receptor tyrosine kinase 2 and neurotrophic receptor tyrosine kinase 3 with large cell‐body diameter, sensory neurons expressing calcitonin gene‐related peptide with medium‐sized cell‐body diameter, and sensory neurons expressing purinergic receptor P2X ligand gated ion channel 3 with small cell‐body diameter.^152^ More recent elegant studies categorized them, by single‐sensory neuron RNA sequencing, into at least 11 distinct neuronal subtypes based on their transcriptomic patterns.^153^, ^154^ Due to the crucial role played by sensory nerves discovered by Hu et al, the question arises as to whether the sensory nerves subpopulations differ in their capacity to regulate BMSC niche.

Importantly, BMSCs are also heterogeneous in their morphology, distribution, anatomical location, origin, molecular markers, and function.^155^, ^156^, ^157^, ^158^, ^159^ At least two subpopulations have been described in the bone marrow.^157^ Thus, whether only a fraction of BMSCs respond to sensory nerves in response to mechanical loading still needs to be elucidated. It would be important to evaluate whether distinct BMSCs' subsets behave differently during sensory nerves activation.

### 2.6 Sensory nerves in the cancer microenvironment

Nerves have been reported to promote tumor growth and spread.^160^, ^161^, ^162^ Classically, sensory nerves have been associated with tumor‐associated pain.^163^, ^164^ Interestingly, a new study shows that sensory nerve fibers are involved in tumor progression in vivo.^124^ The authors show, by using Nav1.8‐Cre/TdTomato mice, the presence of sensory nerves within the tumor microenvironment. Prazeres and colleagues specifically depleted sensory innervations within the tumor microenvironment, in a model of genetic depletion of Nav1.8+ sensory neuronal projections or by chemical depletion using resiniferatoxin. These experiments revealed that sensory nerve ablation induces changes that lead to worse outcomes in tumor‐bearing mice.^124^ Moreover, low expression of genes related to sensory nerves correlate with worse outcomes in human melanoma biopsies.^124^ Taken together, these findings suggest that sensory innervations participate in cancer progression, by inhibiting tumor growth. However, the cellular and molecular mechanisms by which sensory nerves influence cancer development remain to be studied.

**FIGURE 3:**
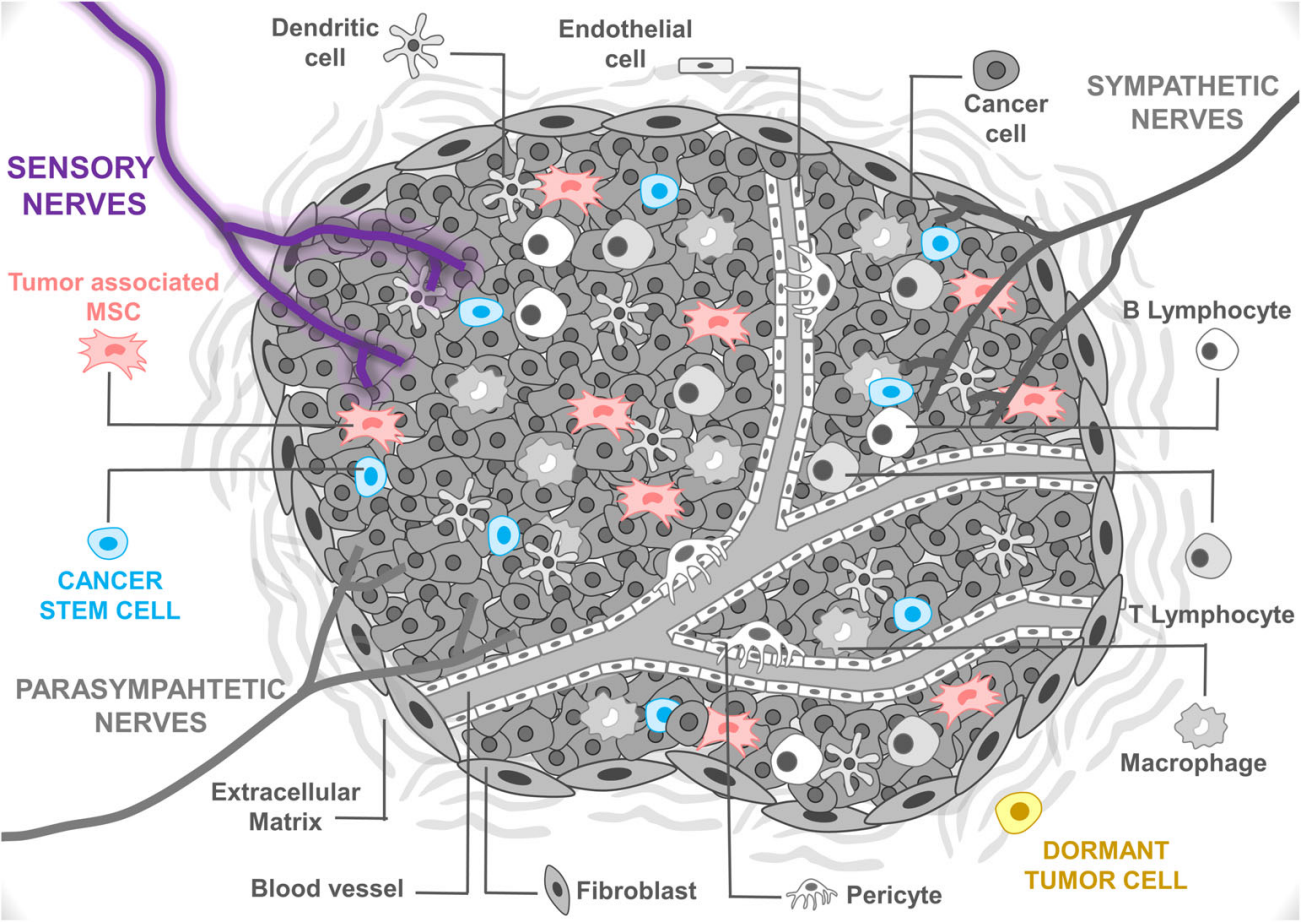
Sensory nerves' possible roles in the tumor microenvironment. Sensory neuronal projections infiltrate the tumor microenvironment. The study of Prazeres and colleagues indicates that sensory nerves block cancer progression.^124^ Cellular and molecular mechanisms by which sensory nerves influence cancer development remain uncertain. Future works should examine whether sensory nerves regulate tumor‐associated mesenchymal stem cells (MSCs), cancer stem cells, or/and dormant tumor cells

Lately, roles in cancer progression have been assigned to tumor‐associated MSCs, which arise as an important component of the tumor microenvironment.^165^ MSCs within the tumors contribute to tumor‐associated immunosuppression, inflammation, angiogenesis, tumor growth, metastasis, and therapeutic resistance in various cancer types.^166^, ^167^ Understanding whether/how tumoral sensory nerves‐derived cues influence MSCs will facilitate our profound comprehension of the functions of sensory nerves and MSCs within the tumor microenvironment. This may lead to the discovery of new cancer therapies that target sensory nerves and MSCs. Not less important will be the exploration of sensory nerves' roles in cancer initiation by cancer stem cells,^168^ or on cancer reactivation by dormant disseminated tumor cells^169^ (Figure 3).

## 3 CONCLUSION

In conclusion, the study by Hu and colleagues reveals a novel important role in BMSCs regulation of sensory neurons which innervate the bone marrow.^92^ However, our understanding of the neural regulation in the stem cell niche in the bone marrow and other organs remains limited, and the complexity of these interactions in different microenvironments should be elucidated in future studies. A big challenge for the future will be to translate these findings to human patients. Whether sensory nerves are essential components in the human bone marrow stem cell niche remains to be determined. Improving the availability of human bone marrow samples will be crucial to reach this goal. Future developments in this research are promising.

## CONFLICT OF INTEREST

The authors declared no potential conflicts of interest.

## AUTHOR CONTRIBUTIONS

All authors wrote and commented on the manuscript.

## Data Availability

Data sharing is not applicable to this article as no new data were created or analyzed in this study.
